# Synthesis and Properties of pH-Thermo Dual Responsive Semi-IPN Hydrogels Based on *N*,*N*’-Diethylacrylamide and Itaconamic Acid

**DOI:** 10.3390/polym12051139

**Published:** 2020-05-16

**Authors:** Huynh Nguyen Anh Tuan, Vo Thi Thu Nhu

**Affiliations:** Faculty of Chemical and Food Technology, HCMC University of Technology and Education, #1, Vo Van Ngan Street, Linh Chieu Ward, Thu Duc District, Ho Chi Minh City 70000, Vietnam; nhuvtt@hcmute.edu.vn

**Keywords:** *N*,*N*’-diethylacrylamide, itaconamic acid, poly(DEA-*co*-IAM), semi-IPN hydrogel, pH-responsive, thermo-responsive, swelling behavior

## Abstract

A series of semi-interpenetrating polymer network (semi-IPN) hydrogels based on *N*,*N*’-diethylacrylamide (DEA) and itaconamic acid (IAM) were synthesized by changing the molar ratio of linear copolymer P(DEA-*co*-IAM) and DEA monomer. Linear copolymer P(DEA-*co*-IAM) was introduced into a solution of DEA monomer to prepare pH-thermo dual responsive P(DEA-*co*-IAM)/PDEA semi-IPN hydrogels. The thermal gravimetric analysis (TGA) revealed that the semi-IPN hydrogel has a higher thermal stability than the conventional hydrogel, while the interior morphology by scanning electron microscopy (SEM) showed a porous structure with the pore sizes could be controlled by changing the ratio of linear copolymer in the obtained hydrogels. The oscillatory parallel-plate rheological measurements and compression tests demonstrated a viscoelastic behavior and superior mechanical properties of the semi-IPN hydrogels. Besides, the lower critical solution temperature (LCST) of the linear copolymers increased with the increase of IAM content in the feed, while the semi-IPN hydrogels increased LCSTs with the increase of linear copolymer content introduced. The pH-thermo dual responsive of the hydrogels was investigated using the swelling behavior in various pH and temperature conditions. Finally, the swelling and deswelling rate of the hydrogels were also studied. The results indicated that the pH-thermo dual responsive semi-IPN hydrogels were synthesized successfully and may be a potential material for biomedical, drug delivery or absorption applications. The further applications of semi-IPN hydrogels are being conducted.

## 1. Introduction

Smart hydrogel material is a three-dimensional network that is able to absorb a large amount of water without dissolving and can undergo a sharp change in volume or phase transition in response to small changes in environmental conditions, such as temperature, pH, ionic strength, light, pressure, redox and electric field [1,2,3,4,5,6,7]. They can be molded into any form, shape or size which is suitable for many potential applications in different fields such as controlled drug delivery [8], smart textile [9], biomedical [10], tissue engineering and regenerative medicine [11]. One of the most popular smart hydrogels is the thermo-responsive polymer materials.

In previous reports, the hydrogels of several *N*-substituted polyacrylamides show thermo-responsive behavior, such as poly(*N*-isopropylacrylamide) (PNIPAM), poly(*N*-cyclopropyl-acrylamide) (PCPA), poly(*N*,*N*’-diethylacrylamide) (PDEA) and poly(*N*-ethylacrylamide) (PEAM) [12,13,14]. These hydrogel materials are capable of reversible phase transition at a certain temperature and is commonly referred to as the lower critical solution temperature (LCST). Below the LCST, the hydrogel is hydrophilic and absorbs water to a swollen state, while above the LCST, on the contrary, it is hydrophobic, and water is expelled from the network to a shrunken state. This phenomenon can be explained by the inter- and intramolecular interaction within the polymer network. At below LCST, strong hydrogen bonds between polymer network and water causes the swollen state, while at above LCSTs, the interaction within polymer network is stronger than the hydrogen bonds, leading to the shrunken state.

Most of the previous studies about thermo-responsive polymer materials were conducted with PNIPAM which displays a LCST at about 32 °C in pure water [15]. Therefore, the characteristics and applicability of polymer materials based on *N*-isoprolylacrylamide (NIPAM) was well understood and contributed to their popularity. However, it was reported that during hydrolysis in aqueous solution, PNIPAM-based materials were able to release toxic low molecular weight amines, then their bio-applications were limited [16]. In this case, the other hydrogels based on PDEA with the LCST of 31 °C [17] can be a new attractive candidate for biomedical applications because the cytotoxicity of PDEA hydrogels is less pronounced than for PNIPAM ones [18]. However, conventional three-dimensional hydrogels also have some disadvantages such as brittle at the room temperature, poor mechanical property, low swelling ratio and slow response rate [19,20]. On the other hand, in a comparison with PNIPAM hydrogels, the swelling ratio of PDEA hydrogels is much lower because of the difference in chemical structure [21]. For several potential applications such as controlled drug delivery or absorbing system, a pH- and thermo- dual responsive hydrogel with a low toxicity, high swelling ratio, fast response rate and good mechanical property is needed because temperature and pH are important environmental factors in these systems. Semi-interpenetrating (semi-IPN) hydrogel based on PDEA, which is prepared by introducing a high hydrophilic linear polymer into the PDEA network, can meet all above requirements. Generally, semi-IPN hydrogels are usually synthesized by simultaneously polymerization of a monomer system with a cross-linking agent in the presence of natural polymer [19,22] or synthetic linear polymer chains [23,24], which will be physically entangled within the polymer network. Besides, a high hydrophilic pH-responsive system can be obtained by introduced a pH-sensitive segment such as acrylic acid [25] into the semi-IPN hydrogel. This way, the resulting hydrogels have good mechanical properties, high response rate, and are also thermo-pH dual-responsive.

In recent years, itaconamic acid (IAM), which has both –COOH and –NH_2_ group, has been used as a pH-sensitive segment to prepare thermo-pH dual responsive copolymers [26,27,28]. In addition, the synthesis of conventional and semi-IPN hydrogels using IAM with significant improvements about mechanical properties, phase transitions and swelling behaviors was also reported [29,30]. In this work, IAM was further used as a co-monomer to synthesize a novel thermo-pH dual responsive semi-IPN hydrogels by free radical polymerization of DEA monomer in the presence of the linear copolymer P(DEA-*co*-IAM) and cross-linker. To the best of our knowledge, this is the first time to combine DEA and IAM together in a semi-IPN hydrogel. FTIR and ^1^H NMR spectra were used to confirm the chemical structures of the polymers, while the GPC and DLS measurements were used to determine some molecular properties. The other properties were further characterized using TGA, DMA, SEM and rheological measurement. Finally, we combined LCST and swelling behavior measurements in various pH buffer solutions (PBS) and different temperatures to investigate the thermo-pH dual responsive of the obtained semi-IPN hydrogels. It is expected that this novel hydrogel has outstanding performances which being more suited to applications in the life sciences or absorption application. The further applications of obtained semi-IPN hydrogels are being conducted by our research group.

## 2. Experimental

### 2.1. Materials

*N*,*N*’-diethylacrylamide (DEA; C_7_H_13_NO) obtained from Tokyo Chemical Industry Co., Ltd. (Japan). Itaconamic acid (IAM; C_5_H_7_NO_3_) was prepared in our laboratory according to the method disclosed in US patent No. 2013/0172490 [31]. Ammonium persulfate (APS; (NH_4_)_2_S_2_O_8_) as an initiator was purchased from Aencore Chemical PTY. Ltd. (Surrey Hills, Australia). *N*,*N*,*N*’,*N*’-tetramethylethylenediamine (TEMED; C_6_H_16_N_2_) as a catalyst and *N*,*N*’-methylenebisacrylamide (MBA, C_7_H_10_N_2_O_2_) as a cross-linker were purchased from Alfa Aesar Co. (Tewksbury, MA, USA). *N*,*N*-Dimethylformamide (DMF) obtained from Macron Fine Chemicals. Deuterium oxide (D_2_O) obtained from Sigma Aldrich (Saint Louis, MO, USA). The buffer solution of pH 4, pH 7 and pH 10 obtained from J. T. Baker Chemical Corp. (Center Valley, PA, USA), the buffer solution of pH 5 obtained from Alfa Aesar Co. (Tewksbury, MA, USA), the buffer solution of pH 6 and pH 8 obtained from Sigma Aldrich (Saint Louis, MO, USA). All reactants were used as received without any further purification.

### 2.2. Preparation of Linear Copolymers P(DEA-co-IAM)

**Figure 1:**
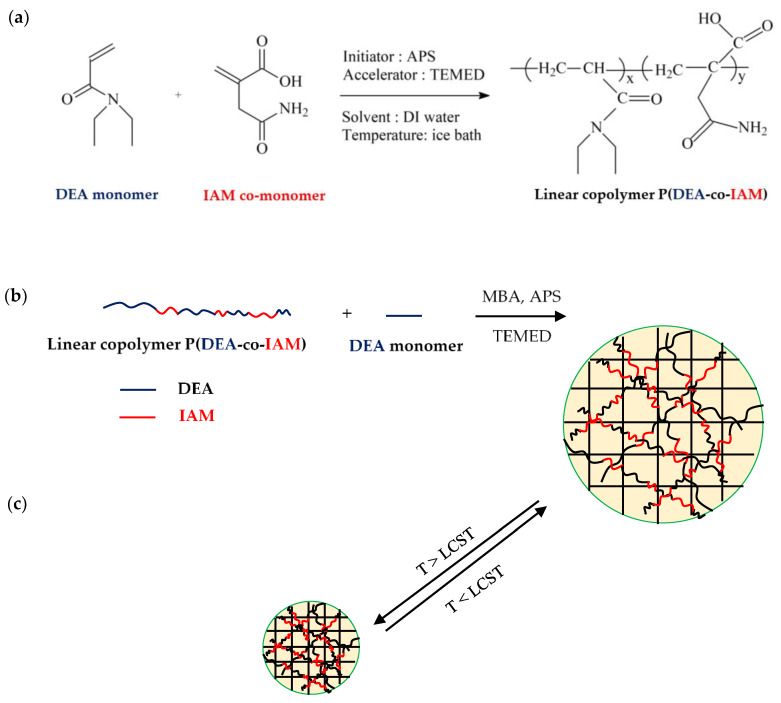
Scheme showing the preparation of: (**a**) linear copolymer P(DEA-*co*-IAM); (**b**) P(DEA-*co*-IAM)/PDEA semi-IPN hydrogel and (**c**) the process of coil-to-globule transition at below and above LCST of semi-IPN hydrogel.

Linear copolymer P(DEA-*co*-IAM) was prepared by free radical polymerization in aqueous solution of DEA and IAM. APS and TEMED were used as an initiator and accelerator, respectively. The preparation is shown in Figure 1a. First, *N*,*N*’-diethylacrylamide (DEA, 2.0 g, 15.72 mmol), itaconamic acid (IAM, with molar ratio of DEA/IAM = 100/1, 100/2, 100/3, 100/4 and 100/5, that is, 0.157; 0.314; 0.471; 0.628 and 0.786 mmol) and 12.0 mL of deionized (DI) water were mixed together in an one-neck flask by continuous stirring under a nitrogen atmosphere until homogeneous, and placed in an ice-water bath. Then, 2.0 mL aqueous solution of APS 0.084 M and 4.0 mL aqueous solution of TEMED 1.067 M were used to initiate the reaction. The obtained linear copolymer P(DEA-*co*-IAM) was ready for the subsequent processes after the polymerization in ice-water bath was carried out for 4 h and stabilized about 24 h at room temperature. To purify the obtained copolymer solution for further investigation, about 2.0 mL of it was dialyzed against DI water using a dialysis membrane with molecular weight cut off is 10,000 g/mol for 7 days (refreshed every half day). Finally, the dried copolymer was received using a vacuum dryer at −50 °C. White solid products were obtained as samples I-1, I-2, I-3, I-4, I-5 for molar ratios of DEA/IAM = 100/1, 100/2, 100/3, 100/4 and 100/5, respectively. For comparison, the linear homopolymer PDEA also was prepared by the same method as described above and named as I-0 sample.

### 2.3. Preparation of Semi-IPN Hydrogels

Semi-IPN hydrogels were prepared by free radical polymerization of DEA monomer in the presence of linear copolymer P(DEA-*co*-IAM) solution and MBA as cross-linker with various designated ratios. APS was used to initiate and TEMED was used to accelerate the reaction. The preparation is shown in Figure 1b. The composition of the hydrogels prepared in this work is summarized in Table 1. For the preparation, linear copolymer P(DEA-*co*-IAM) I-2 solution, DEA monomer, MBA cross-linker and water were mixed together in ice-water bath for 1 h, under nitrogen atmosphere until homogeneous. After that, initiator and accelerator were added, and the solution was mixed together for 3 min. Then, the solution was quickly poured into a cylindrical glass mold with the diameter of 8 mm, sealed and kept at room temperature for 24 h for the polymerization occurred completely. The obtained hydrogels were removed from the molds, cut into samples with the thickness of 2.5 mm and placed in DI water at room temperature in order to eliminate unreacted materials. The DI water was refreshed several times in about one week. Finally, they were freeze-dried at −50 °C and white freeze-dried hydrogel samples were ready for further investigation.

### 2.4. Characterization of the Linear Polymers

Fourier transform infrared (FTIR) spectra were carried out by Perkin Elmer Spectrum RXI FTIR instrument (Waltham, MA, USA) within 4000–400 cm^−1^ having scan resolution of 4.00 cm^−1^. Background measurement were performed and subtracted for all the samples. Proton nuclear magnetic resonance (^1^H NMR) spectra were recorded on a Bruker Advance 300MHz NMR spectrometer (Billerica, MA, USA) at room temperature using D_2_O as the solvent.

Hydrodynamic radii of the linear polymers were confirmed by dynamic light scattering (DLS, Malvern 1000 HAS, Malvern, UK). The 0.5 wt % aqueous solutions of samples were measured at 25 and 45 °C.

The number average molecular weight (*M*_n_) and polydispersity index (PDI) of the linear polymers were determined by gel permeation chromatography (GPC) using a Viscotek GPC system from Malvern Ltd. (Malvern, UK) with DMF as the mobile phase and polystyrene as the standard. About 15 mg of dried sample were dissolved in 5 mL of DMF under following conditions: column 300 mm × 8 mm, flow rate of 1 mL/min, temperature of the column and detector were set to 60 °C, injection quantity of test sample and standard was 150 μL each.

The phase separation profiles and lower critical solution temperatures (LCST) were determined from the transmittance of the sample containing PDEA linear homopolymer or P(DEA-*co*-IAM) copolymers as a function of temperature using a laser transmittance meter (LASOS LGK 7628). The 1.0 wt % aqueous solutions of linear polymer samples were heated from 25 to 50 °C at a heating rate of 1 °C/min. All measurements were taken at a wavelength of 500 nm. The LCST was determined to be the temperature when the transmittance of the solution is 50%.

### 2.5. Characterization of the Hydrogels

A differential scanning calorimeter (DSC 8000, Perkin Elmer, Waltham, MA, USA) was used at a heating rate of 1 °C/min in the temperature range of 20–50 °C to confirm the lower critical solution temperature (LCST) of the swelling hydrogels. The temperature at endothermic peak of DSC trace was referred as LCST of the corresponding hydrogel sample.

A thermal gravimetric analyzer (TGA, Netzsch, TG 209 F3, Netzsch, Selb, Germany) was used at a heating rate of 10 °C/min in the temperature range of 50–600 °C under a nitrogen atmosphere to investigate thermal stability of the semi-IPN hydrogels. The dried samples of linear copolymer P(DEA-*co*-IAM), conventional PDEA and semi-IPN hydrogels were tested.

The SEM images of the freeze-dried hydrogels were taken by a JEOL JSM-7610F scanning electron microscope (Hitachi, Tokyo, Japan) to investigate the interior morphology. All samples were sputter-coated with gold for 10 min to enhance the conductivity and SEM images were acquired at an accelerating voltage of 15.0 kV. The pore sizes and standard deviations were analyzed by ImageJ.

The storage modulus (G’) and loss modulus (G”) were measured to investigate the rheological properties of the swollen hydrogels. Anton Paar MCR 301 rheometer (Anton Paar, Graz, Austria) was used for oscillatory parallel-plate rheological measurements. The G’ and G” were measured as function of time (in the range of 10–500 s, 1 Hz), frequency (in the range of 0.1–10 Hz) and temperature (in the range of 25–50 °C, 1Hz). All tests were performed at a strain of 1%.

The stress-strain curves were tested to investigate the mechanical properties of the hydrogels by a dynamic mechanical analyzer (DMA 7e, Perkin Elmer, Waltham, MA, USA) at room temperature. The compression tests were carried out on the cylindrical samples (Φ10 mm × 5 mm) at a rate of 100 mN/min until cracking. The compressive modulus, fracture strain and fracture stress were confirmed from the stress–strain curves. All tests were performed three times.

### 2.6. Swelling Behavior Study

#### 2.6.1. Swelling Kinetics Measurement

The swelling kinetics of the hydrogels were studied using gravimetric method. The freeze-dried hydrogels were immersed in DI water at 20 °C. At predetermined time points, swelling hydrogels were eliminated the excess water on the surface by filter paper and weighed by an electronic balance. The swelling ratio (SR) was calculated according to the following equation:

(1) $$\mathrm{SR}=\frac{W_{s}-W_{d}}{W_{d}}$$

where *W*_s_ is the weight of the swelling hydrogel and *W*_d_ is the weight of the freeze-dried hydrogel.

#### 2.6.2. Thermo-sensitive Swelling Property

The thermo-sensitive property of the hydrogels was investigated by calculation the equilibrium-swelling ratio as a function of temperature. The freeze-dried hydrogels were immersed in DI water at different temperature points in the range of 20–50 °C until equilibrium. The swelling hydrogels were eliminated the excess water on the surface by filter paper and weighed by an electronic balance, and SR was calculated using the above equation.

#### 2.6.3. pH-Sensitive Swelling Property

The pH-sensitive property of the hydrogels was investigated by calculating the equilibrium-swelling ratio as a function of the pH values. The freeze-dried hydrogels were immersed in pH buffer solution with different pH values in the range of 4–10 at 25 °C until equilibrium. The swelling hydrogels were eliminated the excess water on the surface by filter paper and weighed by an electronic balance, and SR was also confirmed using the above equation.

#### 2.6.4. Deswelling Kinetics Measurements

To investigate the deswelling kinetics of the hydrogels, the freeze-dried samples were firstly immersed in DI water at 25 °C. After one week, the equilibrium swollen hydrogels were quickly transferred to DI water at 37 °C. At predetermined time points, the shrunk hydrogels were removed, wiped off water on the surface by filter paper and weighed by an electronic balance. Water retention (WR) was calculated according to the following equation:

(2) $$\mathrm{WR}(\%)=\frac{W_{t}-W_{d}}{W_{e}-W_{d}}\times 100$$

where *W*_t_ is the weight of the shrunk hydrogel at the specific time *t* at 37 °C, *W*_d_ is the weight of the freeze-dried hydrogel and *W*_e_ is the weight of the equilibrium swollen hydrogel at 25 °C.

## 3. Results and Discussion

### 3.1. Preparation of Semi-IPN Hydrogels

The P(DEA-*co*-IAM)/PDEA semi-IPN hydrogels were synthesized by free radical polymerization of DEA monomer in the presence of linear copolymer P(DEA-*co*-IAM) using MBA, APS and TEMED as cross-linker, initiator and accelerator, respectively. In aqueous solution, the polymerization was initiated by sulfate free radicals, which were produced by the reaction between APS and TEMED [32]. Finally, a three-dimensional network of PDEA was obtained with the linear copolymer P(DEA-*co*-IAM) chains were kept and entangled within the hydrogel network through hydrogen bonding interaction.

### 3.2. ^1^H NMR Measurement

**Figure 2:**
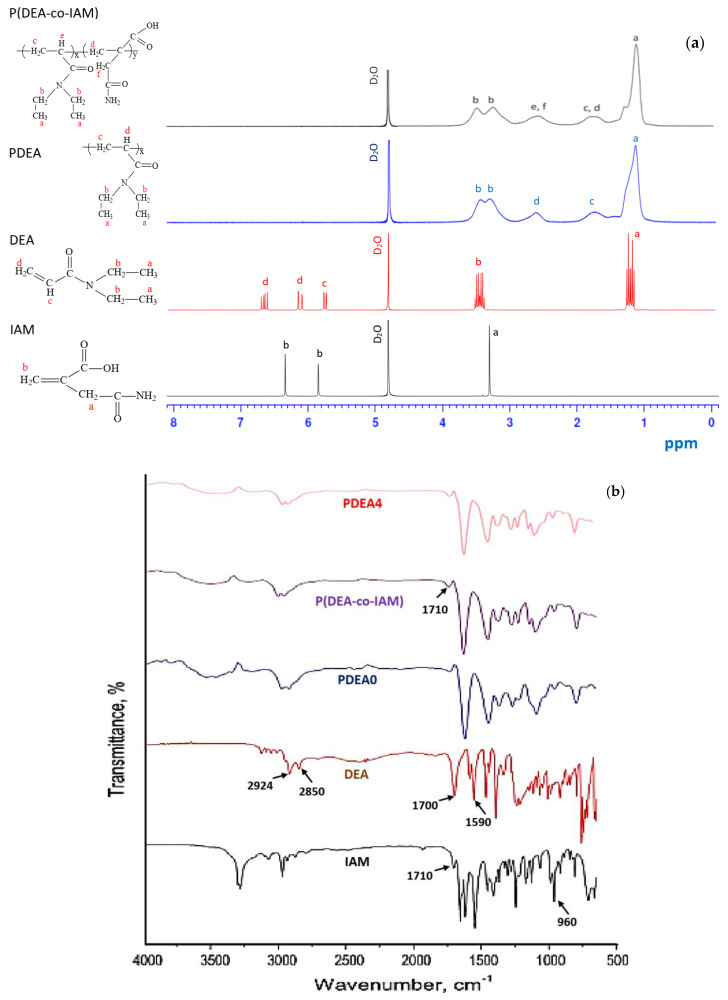
(**a**) ^1^H NMR and (**b**) FTIR spectra of monomers, linear polymers and semi-IPN hydrogel.

Figure 2a shows ^1^H NMR spectra of DEA monomer, IAM comonomer, linear homopolymer PDEA and linear copolymer P(DEA-*co*-IAM). The characteristic peaks from IAM were at δ = 3.3 ppm (–CH_2_–) and δ = 5.9–6.4 ppm (CH_2_=). The characteristic peaks from DEA were at δ = 0.9–1.4 ppm (–CH_3_, in ethyl group); δ = 3.3–3.5 ppm (–CH_2_–, in ethyl group); δ = 5.7 (=CH–) and δ = 6.1–6.7 ppm (CH_2_=). The peaks from DEA at δ = 5.7, 6.1 and 6.7 ppm and from IAM at δ = 5.9 and 6.4 ppm were absent in the spectra of PDEA and P(DEA-*co*-IAM) while the strong bands at 1.1 and 3.3 ppm correspond to CH_3_ and CH_2_ protons of ethyl groups in the side chain; the bands at 1.7 and 2.55 ppm correspond to CH_2_ and CH protons of the main chain appeared indicating successful synthesis of linear homopolymer PDEA and copolymer P(DEA-*co*-IAM) [18,25,33].

### 3.3. FITR Measurement

The FTIR spectra of DEA monomer, IAM comonomer, linear copolymer P(DEA-*co*-IAM), conventional PDEA and semi-IPN hydrogels are shown in Figure 2b. In the spectrum of DEA monomer, the amide C=O stretching peak was shown at about 1700 cm^−1^, the C–H stretching vibration band of –CH_3_ and –CH_2_– groups were shown at about 2924 and 2850 cm^−1^, and the C=C stretching peak was shown at 1590 cm^−1^ [34]. In the spectrum of IAM monomer, the C=C peak was shown at 960 cm^−1^ and had a distinct characteristic peak at 1710 cm^−1^ which corresponds to the C=O peak in carboxylic group [28]. After polymerization, the spectrum of linear copolymer P(DEA-*co*-IAM), conventional PDEA and semi-IPN hydrogels showed an absence of the peak at about 960 and 1590 cm^−1^, indicating C=C was transformed into C–C. Moreover, the C–H stretching vibration band of –CH_3_ and –CH_2_– groups for DEA monomer were also detected for conventional PDEA and semi-IPN hydrogels. In addition, the band at 1710 cm^−1^ due to the stretching of C=O bond indicates the presence of IAM in the linear copolymer and semi-IPN hydrogel. Thus, the FTIR spectra showed successful synthesis of the linear copolymer P(DEA-*co*-IAM), conventional PDEA and semi-IPN hydrogels.

### 3.4. Dynamic Light Scattering (DLS) and Gel Permeation Chromatography (GPC)

**Figure 3:**
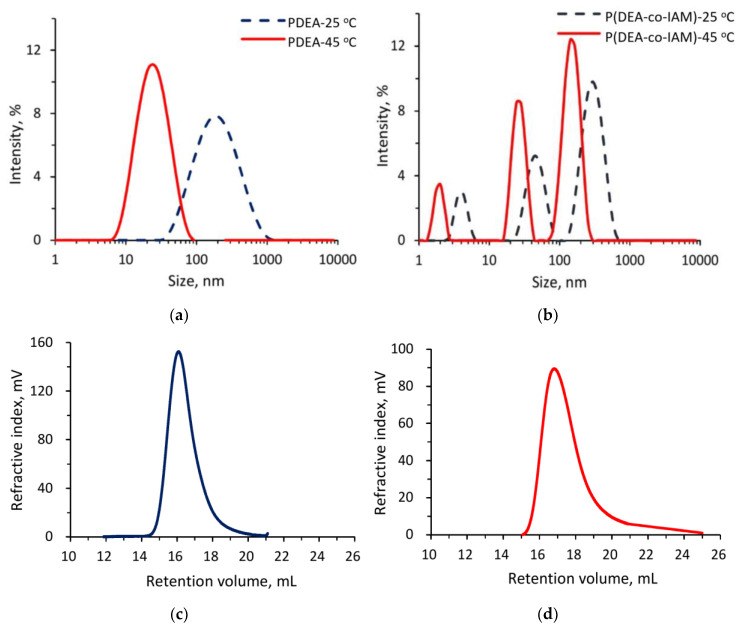
DLS of (**a**) linear homopolymer PDEA and (**b**) linear copolymer P(DEA-*co*-IAM) at 25 (below LCST) and 45 °C (above LCST); (**c**) GPC trace of linear homopolymer PDEA and (**d**) linear copolymer P(DEA-*co*-IAM).

The temperature-induced transition of linear polymers was investigated by dynamic light scattering (DLS). The DLS results of aqueous linear homopolymer PDEA and linear copolymer P(DEA-*co*-IAM) solutions at below and above LCST are shown in Figure 3a,b. For linear homopolymer, DLS showed one size distribution with a hydrodynamic diameter (*D*_H_) of around 190.0 and 24.4 nm at 25 and 45 °C, respectively. On the contrary, the polydispersion was observed in the DLS of linear copolymer suggesting the presence of small oligomers and some swollen aggregates of large size (in the range of 4.0 to 295.0 nm and of 2.0 to 164.0 nm at 25 and 45 °C, respectively). These results can be explained by the influence of IAM monomer. In DI water, the IAM fraction within the linear copolymer P(DEA-*co*-IAM) could be ionized resulting the charged copolymer chains. Therefore, the aggregation of charged copolymer chains was much easier than that of homopolymer. The same results were noted in the study by Hayde et al. [35] and Andre et al. [36]. On the other hand, as the temperature increased from 25 to 45 °C, the *D*_H_ of polymer and copolymer decreased dramatically because the coil-to-globule transition behavior occurred revealing a typical thermally induced aggregation [37].

**Figure 4:**
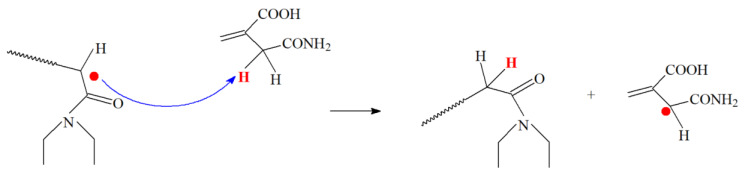
The chain termination of free radical polymerization.

Gel permeation chromatography (GPC) was used to investigate the influence of IAM monomer to the number average molecular weight (*M*_n_) and polydispersity index (PDI) of linear polymers. Figure 3c,d show the GPC curves of the homopolymer PDEA and linear copolymer P(DEA-*co*-IAM), respectively, while their *M*_n_ and PDI are shown in Table 2. The PDI was rather narrow with the value of 1.68 and not significantly affected by IAM monomer. The *M*_n_ of P(DEA-*co*-IAM) (5.80 × 10^4^ g/mol) was lower than PDEA (11.5 × 10^4^ g/mol). It may be the influence of methanediyl group (–CH_2_–) from IAM composition units. In the propagation of free radical polymerization, the unpaired electron at the end of a growing chain could be paired with an electron of carbon-hydrogen bond from methanediyl group of IAM leading to the chain termination occurred quickly. This process is illustrated in the Figure 4. Besides, the GPC curve of linear copolymer P(DEA-*co*-IAM) showed one size distribution, while the DLS results presented a polydisperse with three peaks. The main factor explaining this difference could be temperature. As mentioned above, the hydrodynamic radii of linear polymers were measured at 25 and 45 °C, while the molecular weights were confirmed at 60 °C. At high temperatures, the ionization of the IAM segments within linear copolymer P(DEA-*co*-IAM) took place stronger, so the oligomers could be aggregated together more easily to form the larger globules.

### 3.5. Thermal Gravimetric Analyses (TGA)

**Figure 5:**
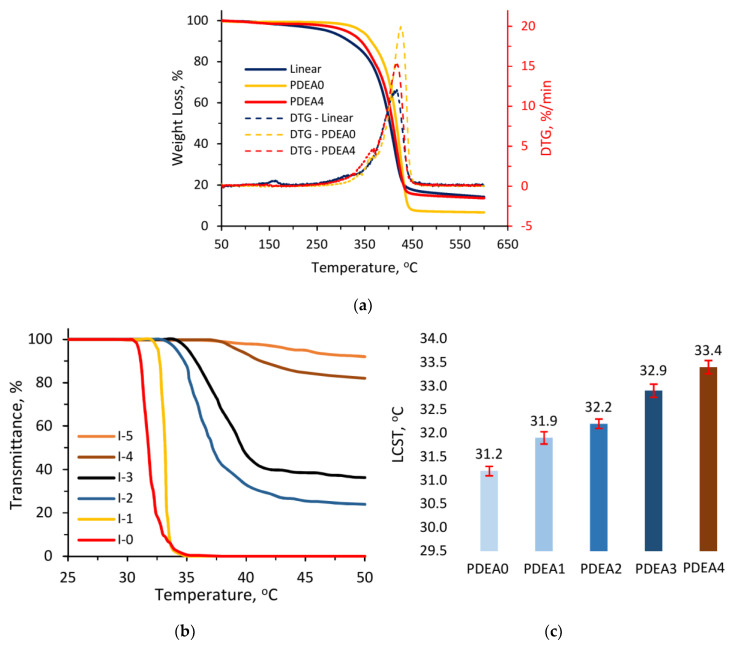
(**a**) TGA and DTG curves of linear copolymer P(DEA-*co*-IAM), conventional PDEA and semi-IPN hydrogels; (**b**) the phase transition curves of linear copolymer P(DEA-*co*-IAM) aqueous solutions; and (**c**) the LCSTs which were determined by DSC of equilibrium swollen hydrogels in DI water.

Thermogravimetric analysis (TGA) was carried out to investigate the thermal stability of the hydrogel materials. As shown in Figure 5a, the linear copolymer P(DEA-*co*-IAM) had a small weight loss of 2% at 170 °C, then the weight loss began to decline rapidly in the range of 260–430 °C and remaining weight was observed about 14% at 600 °C. This result could be explained by the degradation of IAM segments and the rest of these groups linked to DEA, respectively. The same results were reported by Sousa et al. for poly(*N*-isopropylacrylamide-*co*-acrylamide) gels [38] and Rwei et al. for P(NIPAM-*co*-IAM) [30]. For conventional PDEA hydrogel, the decomposition of polymer network occurred from 310 to 440 °C. Then, the thermal degradation was almost unchanged from 440 to 600 °C with the remaining weight of 6.7%. In case of semi-IPN hydrogel, the thermal degradation took place in two stages. The first stage ended at 160 °C with a very small weight loss about 1.5% which was attributed to the depolymerization of IAM segments. The second stage started at about 280 °C, which was between the P(DEA-*co*-IAM) and PDEA hydrogel proving a strong interaction between the linear copolymer and hydrogel network via hydrogen bonds [39]. Moreover, the thermal stability of semi-IPN hydrogel was improved with a residual mass of 13.6% at 600 °C which was higher than the conventional PDEA hydrogel at the same temperature. In comparison with other semi-IPNs based on PDEA, Wei et al. reported that the residual mass of salecan/poly(*N*,*N*-diethylacrylamide-*co*-methacrylic acid) semi-IPN hydrogel was 13%, which was lower than our current work [22]. This may be due to the linear copolymer P(DEA-*co*-IAM) possessed higher hydrophilicity than salecan molecules leading to stronger hydrogen bonds in the P(DEA-*co*-IAM)/PDEA semi-IPN hydrogel. 

### 3.6. Lower Critical Solution Temperature (LCST)

For linear polymers, the phase transition behavior is shown in Figure 5b. As clearly seen, the phase transition curves of I-0 and I-1 samples were sharp, whereas the transmittance decreased gradually with other samples and did not reach 0% in the measured temperature range. The LCSTs of copolymer strongly increased as the IAM molar fraction increased, and the I-0 sample without IAM units possessed the lowest value. Specifically, LCSTs are 31.8, 33.2, 37.2 and 39.5 °C for the I-0, I-1, I-2 and I-3 samples, respectively, while the LCSTs were disappeared for I-4 and I-5 samples, indicating the incorporation of hydrophilic components IAM might decrease the thermo-sensitivity of the resulted copolymer P(DEA-*co*-IAM). Such a phenomenon may be because the hydrophilic groups, such as carboxyl and amino groups in the copolymers, which originated from IAM formed hydrogen bonds with water to increase the LCST. That is, there are more hydrophilic groups in the copolymer than the homo-polymer, and the addition of IAM will significantly increase the LCSTs of obtained copolymers. Besides, the ionization of high hydrophilic units inside the copolymer may interfered in the thermally induced phase separation due to the ionic stabilization [40]. In this work, after considering between the thermal sensitivity and swelling behavior factors, we decided to use the I-2 sample as a linear copolymer for the synthesis of semi-IPN hydrogels.

For the hydrogels, LCST also is the most important factor demonstrating the thermo-sensitive behavior. In this work, the LCSTs of the hydrogels were measured by DSC analyses and shown in Figure 5c. The conventional PDEA hydrogel had the lowest LCST of 31.2 °C. In comparison with linear homopolymer PDEA, conventional hydrogel showed a shift of LCST to a lower value. This phenomenon was ascribed to the lower mobility of three-dimensional network than the linear structure [33]. For semi-IPN hydrogels, LCSTs shifted from 31.9 to 33.4 °C depending on the volume of linear copolymer introduced. This result can also be explained by the formation of hydrogen bonds between water and hydrophilic functional groups such as carboxyl and amino in the linear copolymer P(DEA-*co*-IAM) leading to the increase of the LCST. Similar results were also observed in the previous reports not only for linear copolymers but also for hydrogel materials [26,27,28,29]. These results clearly demonstrate that the obtained semi-IPN hydrogels are thermo-sensitive.

### 3.7. Interior Morphology

**Figure 6:**
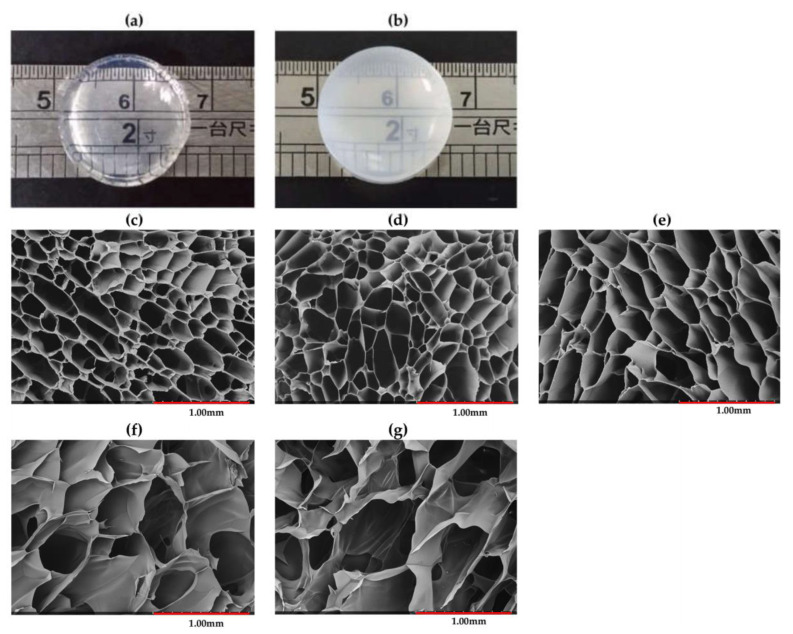
Photograph (**a**–**b**) and SEM images (**c**–**g**) of conventional PDEA and semi–IPN hydrogels: (**a**) PDEA0, (**b**) PDEA2, (**c**) PDEA0, (**d**) PDEA1, (**e**) PDEA2, (**f**) PDEA3 and (**g**) PDEA4.

The photographs of conventional and semi-IPN hydrogel (PDEA2) are described in Figure 6a,b, respectively. The semi-IPN hydrogels were opaque and elastic, showing a successful synthesis of the novel hydrogels. SEM images of the freeze-dried hydrogels are presented in the Figure 6c–g indicating a clear porous structure. In the conventional hydrogel network, the thick wall could be observed with the pore size of about 220 ± 77 μm. The thick wall of conventional hydrogels based on DEA was also reported by Zhang et al. [23] and Chen et al. [41].

The SEM images clearly showed the dependence of interior morphology on the linear copolymer content introduced into the semi-IPN hydrogels. As shown in the Figure 6c–g, these hydrogels remained the porous structure with the average pore sizes tended to increase with the increase of the linear copolymer content. Table 3 showed the pore sizes of PDEA1, PDEA2, PDEA3 and PDEA4 sample were 238 ± 78, 282 ± 51, 524 ± 59 and 715 ± 83 µm, respectively. This may be explained by the hydrophilicity of IAM segments and crosslinking density of the samples. According to Table 1, from PDEA1 to PDEA4 samples, the linear copolymer content increased, and MBA content decreased, leading to an increase in hydrophilicity and a decrease in crosslinking density, respectively. It was reported that the lower crosslinking density produced a larger pore size and higher density hydrogel [42,43]. On the other hand, an increase of linear copolymer content gives rise of the absorbed water amount and created larger space inside the hydrogels after freeze-drying. Generally, the interior morphology could be controlled by changing the linear copolymer content introduced into the semi-IPN hydrogels. The similar phenomenon was also reported by Chen et al. [19], Wei et al. [22] and Zhang et al. [23].

### 3.8. Rheological Measurement

**Figure 7:**
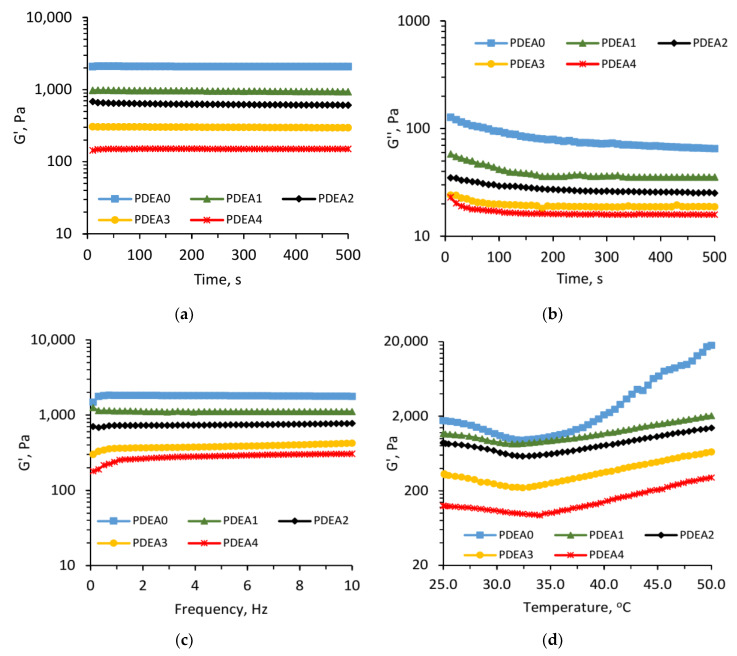
Storage modulus G’ (**a**) and loss modulus G” (**b**) as a function of time; Storage modulus (G’) as a function of frequency (**c**) and temperature (**d**) of the conventional and semi-IPN hydrogels.

The storage modulus (G’) and loss modulus (G”) of the conventional, as well as semi-IPN hydrogels with different feeding ratios were measured by using an oscillatory rheometer. The Figure 7a–c show the G’ values did not depend on time, frequency in the employed range and were much higher than the G” values, indicating a viscoelastic behavior of hydrogel materials [44]. Moreover, it can be observed that G’ and G” values of semi-IPN hydrogels were lower than that of conventional hydrogel. For semi-IPN hydrogels, G’ and G” values decreased with the increase of linear copolymer P(DEA-*co*-IAM) content introduced into the three-dimensional network. This phenomenon can be explained by the crosslinking density and the flexibility of the resulted hydrogels. More linear copolymer in the semi-IPN materials reduced the crosslinking density and enhanced the flexibility of polymer network due to generation of fewer entanglements in the hydrogel structure, leading to a reduction of elastic modulus [22,45].

The influence of temperature on G’ values is shown in Figure 7d. As can be observed, all the hydrogels exhibited the same rheological behavior with temperature. The G’ values decreased slightly in the temperature range of 25–33 °C, while they increased continuously at temperature above 33 °C. Again, these results strongly confirmed the thermal sensitivity of the obtained hydrogels. At below LCST, the oscillation of linear copolymers was increased with increasing temperature, resulting increased flexibility of the overall hydrogel network and reduced the elastic modulus. On the contrary, at above LCST, the absorbed water was expelled from the polymer network due to poor interaction between PDEA and water. Therefore, the crosslinking density increased as well as G’ values increased.

### 3.9. Mechanical Properties.

**Figure 8:**
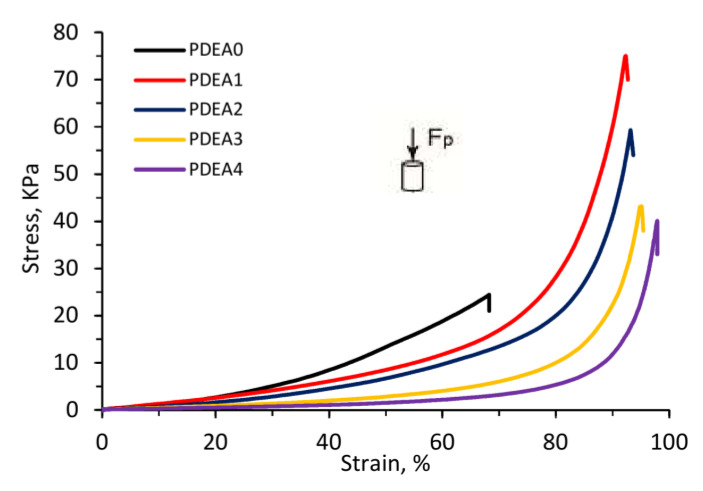
The stress–strain curves of conventional and semi-IPN hydrogels at 25 °C.

Figure 8 shows the stress–strain curves, while the compressive modulus, fracture strain and fracture stress of the hydrogels are listed in Table 3. The semi-IPN hydrogels had lower compressive modulus compared with the PDEA conventional hydrogel. As shown in the Table 1, from PDEA0 to PDEA4 samples, the PDEA content decreased leading to decrease the crosslinking density and hardness of hydrogels, then decreased compressive modulus. Besides, the fracture stress of semi-IPN hydrogels increased significantly compared with that of conventional one. This phenomenon can be explained by the fact that the linear copolymer chains P(DEA-*co*-IAM) within semi-IPN hydrogel acted as a stress-dispersing agent and subjected to higher stress. Similar result was reported by Liu et al. for poly(*N*-isopropylacrylamide-*co*-hydroxyethyl methacrylate)/poly(*N*-isopropylacrylamide) semi-IPN hydrogels [20]. For semi-IPN hydrogels, from PDEA1 to PDEA4 samples, the fracture stress was gradually decreased which was ascribed to the difference of pore sizes. It was reported that the hydrogels with small pore sizes prevented the crack propagation when they were subjected to external forces because of the low stress concentration [46]. Figure 6c–g and Table 3 show PDEA4 sample possessed the biggest pore size and lowest fracture stress. Generally, the stress distribution took place faster and easier in the smaller pore size hydrogel networks. Similar results were found in many semi-IPN hydrogel systems [8,30,47,48].

Another weakness of conventional PDEA hydrogels is low fracture strain compared with that of semi-IPN hydrogels. This can be a significant drawback in reusing a conventional hydrogel for absorption applications. Table 3 shows that the PDEA0 sample had the lowest fracture strain of 68.2%, while the semi-IPN hydrogels had higher values in the range of 92.3–97.9%. Generally, the high molecular weight of P(DEA-*co*-IAM) created a high viscosity medium, which assisted the energy dissipation, took place rapidly during deformation. On the other hand, the semi-IPN hydrogels were high hydrophilic which helped water molecules were kept tighter in the three-dimensional network, leading to increase significantly fracture strain, because water worked as a plasticizer [49]. The similar results can also be found in the literatures [8,30,47,48]. These above results confirmed the excellent mechanical properties of semi-IPN hydrogels compared with that of the conventional hydrogel.

### 3.10. Swelling Behavior

#### 3.10.1. Swelling Kinetics

**Figure 9:**
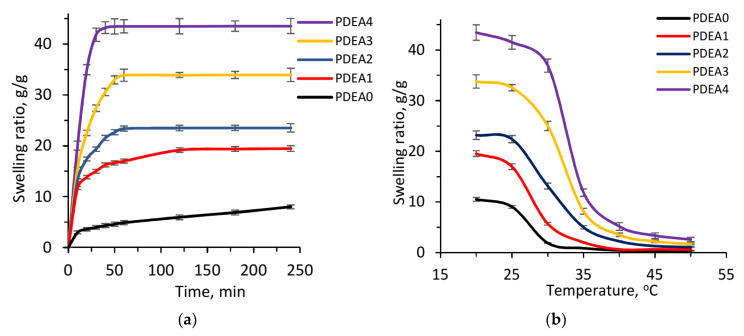

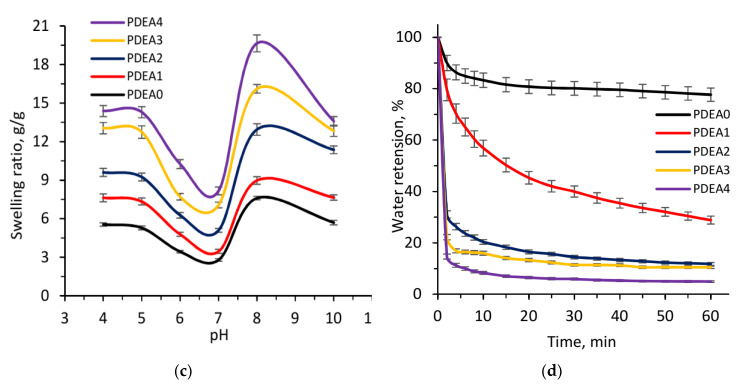
(**a**) Swelling kinetic curves in deionized water at 20 °C; (**b**) equilibrium swelling ratio values in DI water as a function of temperature from 20–50 °C, (**c**) in PBS with pH values range from pH = 4–10 at 25 °C of conventional PDEA and semi-IPN hydrogels; and (**d**) deswelling kinetic curves of hydrogels at the temperature changed from 25 to 37 °C in DI water.

Figure 9a shows the swelling kinetic curves of the dried hydrogels with different feeding ratios in DI water at 20 °C. It was clearly seen that the semi-IPN hydrogels had a faster swelling rate and higher equilibrium-swelling ratio than the conventional hydrogel, and these values were gradually increased as the linear copolymer content increased. For example, the swelling ratio of PDEA0 sample was about 3.60 g/g within 20 min, about 4.89 g/g within 60 min, while this value of PDEA1, PDEA2, PDEA3 and PDEA4 samples were about 13.69, 17.15, 22.25 and 35.08 g/g within 20 min, and about 16.79, 23.03, 33.42 and 43.65 g/g within 60 min, respectively. This result can be attributed by the hydrophilicity of linear copolymer originating from IAM units as discussed above. Besides, it was also believed that the high molecular weight chains of linear copolymer acted as channels for moving water in semi-IPN hydrogel structure [22,50]. Thus, it could assist water molecules move easily from outside into the hydrogel network, leading to increase the swelling rate. On the other hand, it was reported that the swelling kinetic was influenced by the crosslinking density of the polymer networks [21]. In this work, from PDEA0 to PDEA4 sample, the crosslinking density gradually decrease makes PDEA4 sample had the largest pore size, highest swelling rate and highest swelling ratio. This result demonstrated that the swelling kinetics of semi-IPN hydrogels could be controlled by changing the linear copolymer content.

#### 3.10.2. Effect of Temperature

The swelling behavior in DI water at different temperatures was tested to demonstrate the thermo-sensitive property of the obtained hydrogels and the results are shown in Figure 9b. As clearly described, at the same temperature, the equilibrium-swelling ratio of the hydrogels increased from PDEA0 to PDEA4 samples, due to the high hydrophilicity of IAM co-monomer. For example, at 20 °C PDEA0 had the highest swelling ratio of 10.52 g/g, while these values for PDEA1, PDEA2, PDEA3 and PDEA4 samples were 19.55, 23.19, 33.78 and 43.44 g/g, respectively. In the temperature range of 30 to 35 °C, the swelling ratios decreased dramatically. This result is consistent with LCST values as shown in Figure 5c. Generally, the thermo-responsive semi-IPN hydrogel was shrunken at above LCST and swollen at below LCST because it passed through the coil-to-globule transition [17], and was illustrated in Figure 1c. In comparison with other semi-IPN hydrogels based on DEA, such as Chen et al. [19], Wei et al. [22] and Zhang et al. [23], IAM in this work exhibited the remarkable hydrophilicity with the swelling ratio of 43.43 g/g. Moreover, Figure 9b also shows no significant difference in the swelling ratios of the hydrogels at the temperatures above LCST. This can be explained by the collapse of semi-IPN hydrogels to form the same network structures at high temperatures.

On the other hand, when the temperature was increased from 20 to 50 °C (below and above LCST, respectively), the values of reduction in swelling ratio gradually increased from PDEA0 to PDEA4 samples. For example, PDEA0 had the smallest change in the swelling ratio ($\Delta {SR}_{T}{=SR}_{20\;{}^{\circ}C}{-SR}_{50\;{}^{\circ}C})$ of 10.11 g/g, while this value was 40.06 g/g for PDEA4 sample, which ascribed by the difference in the pore sizes and hydrophilicity between the hydrogels [23]. This result demonstrated the superiority of the semi-IPN over conventional hydrogel for smart applications such as drug delivery, absorption and separation processes.

#### 3.10.3. Effect of pH

The swelling behavior in different pH values was tested to demonstrate the pH-responsive properties of the hydrogels. Figure 9c shows the equilibrium swelling ratios of the obtained hydrogels in pH buffer solutions from pH 4 to 10 at 25 °C. In this work, the linear copolymer P(DEA-*co*-IAM) is a kind of polyampholyte, which contains both anionic and cationic groups originating from IAM units. Thus, the dissociation of carboxylic acid and amino groups was strongly influenced by the pH environment, then significant influenced to swelling behavior. It is clearly seen that the swelling ratios of the hydrogels in pH buffer solutions were much lower than those in DI water because these solutions have a high ionic concentration. As shown in Figure 9c, at the same pH values, the swelling ratios of the hydrogels increased as the linear copolymer P(DEA-*co*-IAM) content increased. This can be explained by the high hydrophilic IAM units as discussed above. 

The tendency of swelling ratios in different pH solutions was quite diverse. They were rather high at pH 4, and slightly decreased at pH 5 before reaching the lowest value at pH 7. However, they were raised sharply again at pH 8 and decreased a little at pH 10. This result was also found in semi-IPN hydrogels based on NIPAM and IAM, and was explained by the repulsion of the moieties with the same type of charge in the hydrogel systems [30]. In a neutral media, the electrostatic repulsion disappeared leading to the lowest swelling ratio, while in acid or base media, this electrostatic interaction rapidly expanded hydrogel networks and increased the swelling ratio. The slightly reduction of swelling ratio as the pH value went up from 8 to 10 was explained by the “charge screening effect” [51].

#### 3.10.4. Deswelling Kinetics

For many absorption applications, thermo-responsive hydrogel is expected having not only high response rate but also high deswelling rate. Figure 9d shows the deswelling kinetics of the hydrogels, which was tested by transferring the equilibrium swollen hydrogels in DI water from 25 °C (below LCST) to 37 °C (above LCST). In Figure 9d, at the same testing time, the water loss of the hydrogels increased as the linear copolymer content increased. For example, PDEA0 sample lost only 16.17% water within 10 min, while PDEA1, PDEA2, PDEA3 and PDEA4 sample lost 43.11%, 79.64%, 84.04% and 91.68%, respectively. Generally, a thermo-responsive hydrogel network shrinks and releases absorbed water from the polymer network at above LCSTs. When the conventional hydrogel PDEA0 was immersed into water at 37 °C, the shrinkage took place and formed a dense skin layer on the surface of material. This dense skin layer locked the surface of hydrogel so that the released water could not move out leading to a slow deswelling rate. For the semi-IPN hydrogels, the hydrophilic linear copolymer provided many releasing channels [50], which prevented the formation of dense skin layer, so the water molecules could be moved out easily when their structure were collapsed at 37 °C. The more releasing channels were formed the more linear copolymers were introduced, leading to an increase in deswelling rate. This can be used to design a thermo-responsive hydrogel with some desired properties.

## 4. Conclusions

In this work, a series of thermo-pH dual responsive semi-IPN hydrogels based on DEA and IAM were prepared by the free radical polymerization of DEA aqueous solutions in the presence of the linear copolymer P(DEA-*co*-IAM) and cross-linker MBA. IAM co-monomer reduced the average molecular weight of the linear copolymer, and disappeared at the one-size distribution of the hydrodynamic diameter. Characteristics of the linear copolymer and the hydrogels were investigated by FTIR, ^1^H NMR, TGA, DSC, SEM, rheological and compressive tests. The chemical structure was confirmed by FTIR and ^1^H NMR spectra, and suggests that the linear polymer chains were entangled in the three-dimensional network. TGA and mechanical tests revealed the thermal stability and good mechanical properties of the semi-IPN hydrogels. Moreover, the viscoelastic behavior of the obtained hydrogels was also confirmed by rheological tests with the G’ values were much higher than G”. SEM images showed a porous structure of the hydrogels with pore size in the range from 220 ± 77 to 715 ± 83 µm. The LCST of the semi-IPN hydrogels in DI water increased slightly from 31.3 to 33.4 °C as increased the linear copolymer content. The thermo-pH dual responsive of semi-IPN hydrogels could be presented by their swelling behavior in different temperatures and pH buffer solutions: increased linear copolymer content or acid/base solution increased the swelling ratio, and this value dropped rapidly as the temperature increased above LCST. Finally, the swelling and deswelling rates of the semi-IPN hydrogels were also increased quickly with the increase of the linear copolymer content. Thus, the properties of semi-IPN hydrogels absolutely could be controlled by changing the ratio of the linear copolymer introduced into the PDEA network. The phase transition and swelling behavior of the semi-IPN network in this work can be significantly changed by a change of temperature and/or the pH environment, indicating it can be used for a drug delivery system or as a material to absorb heavy metal ions. Further applications of semi-IPN hydrogels based on DEA and IAM are in progress.

## Figures and Tables

**Table 1:** Composition of conventional and semi-IPN hydrogels.

Samples	Linear Copolymer P(DEA-*co*-IAM), I-2 Solution (mL) ^a^	DEA, (mL)	DI water (mL)	MBA (mg)	APS ^b^ Solution (mL)	TEMED ^c^ Solution (mL)	Total Volume (mL)
PDEA0	0	1.50	9.0	46.0	1.5	3.0	15
PDEA1	1.5	1.35	8.0	41.0	1.4	2.8	15
PDEA2	3.0	1.20	7.2	37.0	1.2	2.4	15
PDEA3	4.5	1.05	6.2	32.0	1.1	2.2	15
PDEA4	6.0	0.90	5.4	28.0	0.9	1.8	15

^a^ Solution of 1 g/10 g DI water; ^b^ solution of 0.4792 g APS/25 mL DI water; ^c^ solution of 4 mL TEMED/25 mL DI water.

**Table 2:** Molecular weight and polydispersity index (PDI) of linear polymer/copolymer.

Samples	*M*_n_ × 10^4^, g mol^−1^	PDI
Linear copolymer P(DEA-*co*-IAM)	5.80	1.68
Linear homopolymer PDEA	11.5	1.68

**Table 3:** Compressive properties of hydrogels.

Sample	Compressive Modulus (kPa)	Fracture Strain (%)	Fracture Stress (kPa)	Pore Size (μm)
PDEA0	3.4 ± 0.5	68.2 ± 2.9	24.4 ± 2.0	220 ± 77
PDEA1	1.9 ± 0.3	92.3 ± 4.8	75.0 ± 3.7	238 ± 78
PDEA2	1.7 ± 0.3	93.2 ± 5.2	59.3 ± 4.1	282 ± 51
PDEA3	0.6 ± 0.2	95.1 ± 4.3	43.1 ± 3.2	524 ± 59
PDEA4	0.3 ± 0.1	97.9 ± 3.8	40.0 ± 2.4	715 ± 83
